# Determining the protective characteristics and risk factors for the development of anastomotic leakage after low anterior resection for rectal cancer

**DOI:** 10.1007/s00595-020-02133-0

**Published:** 2020-10-02

**Authors:** Nobuaki Suzuki, Shin Yoshida, Shinobu Tomochika, Yuki Nakagami, Yoshitaro Shindo, Yukio Tokumitsu, Michihisa Iida, Shigeru Takeda, Shoichi Hazama, Tomio Ueno, Hiroaki Nagano

**Affiliations:** 1 Department of Gastroenterological, Breast and Endocrine Surgery, Yamaguchi University Graduate School of Medicine, Ube, Japan; 2 Department of Translational Research and Developmental Therapeutics Against Cancer, Yamaguchi University School of Medicine, Ube, Japan; 3 Department of Digestive Surgery, Kawasaki Medical School, Kurashiki, Japan

**Keywords:** Colorectal surgery, Anastomotic leak, Risk factor, Rectal cancer, Pelvic index

## Abstract

**Purpose:**

Anastomotic leakage is one of the most serious postoperative complications associated with surgery for rectal cancer. The present study aimed to identify the protective characteristics and risk factors associated with anastomotic leakage after low anterior resection for rectal cancer.

**Methods:**

This was a retrospective, single-center study conducted between January 2009 and December 2017 at our institution. In total, 136 rectal cancer patients who underwent low anterior resection were included in the study. We analyzed preoperative and intraoperative factors. In addition, the pelvic dimensions were measured using computed tomography in all cases.

**Results:**

Among the 136 patients, anastomotic leakage occurred in 21 (15.4%), including 18 males and 3 females. The median body mass index was 21.1 kg/m^2^. The construction of a covering stoma was found to be a protective factor. In addition, the operation time (≥ 373 min), intraoperative blood loss (≥ 105 ml), and size of the pelvic inlet (≥ 113 mm) were identified as risk factors for anastomotic leakage.

**Conclusion:**

The construction of a covering stoma was a possible protective factor. However, a longer operation time, higher intraoperative blood loss, and larger pelvic inlet dimensions were possible risk factors for developing anastomotic leakage after low anterior resection in patients with rectal cancer.

## Introduction

Advances in surgical procedures and adjuvant therapies have made sphincter-preserving surgery the standard operation for most patients with rectal cancer. Heald et al. introduced a new method called total mesorectal excision (TME) for the treatment of rectal cancer. TME as a novel surgical method is important for preventing injury to the fascia propria of the rectum. At present, this technique is considered the gold standard for managing rectal cancer surgery [1–3].

Anastomotic leakage (AL) is a postoperative complication that occurs in patients who undergo low anterior resection (LAR) for rectal cancer [4–6]. AL leads to several serious postoperative complications, including peritonitis, sepsis, need for re-operation or percutaneous intervention, prolonged hospitalization, increased medical costs [7–9], and a poor prognosis [10, 11]. The basic requirements for anastomotic healing are proper healthy bowel ends and tension-free anastomosis [12]. Previously reported risk factors for AL during surgery include the operation time, amount of intraoperative blood loss, and blood transfusion [13, 14]. Tsuruta et al. reported that a smaller ratio of the difference between the interspinous distance and diameter of the mesorectum to the depth of the lesser pelvic cavity (pelvic index) was associated with a higher risk of AL [15]. Similarly, a few studies have reported the detection of risk factors for AL using preoperative imaging findings [16, 17]. However, there is currently no consensus regarding the risk factors associated with AL.

The present study, therefore, explored the protective as well as risk factors for AL by analyzing preoperative and intraoperative features on preoperative reconstructed coronal and sagittal computed tomography (CT) images.

## Methods

### Study population

**Fig. 1:**
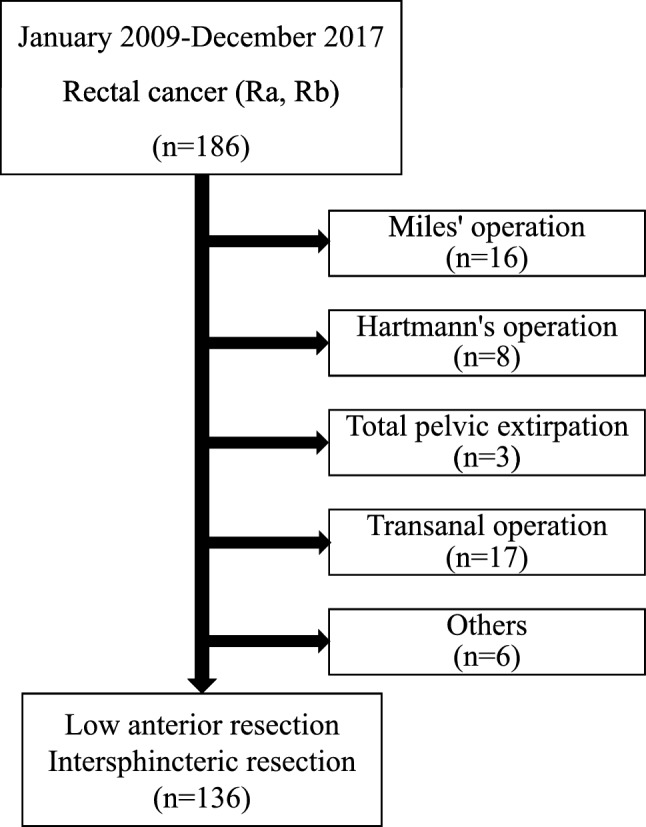
CONSORT diagram. This was a retrospective, single-institution study of 136 patients with rectal cancer (Ra, Rb) who underwent low anterior resection with the double stapling technique or handsewn anastomosis between January 2009 and December 2017 at our institution

A total of 186 patients with rectal cancer who consecutively underwent surgery at the Department of Gastroenterological, Breast and Endocrine Surgery, Yamaguchi University Graduate School of Medicine, between January 2009 and December 2017, were enrolled. Among those patients, 50 were excluded, because they underwent surgery using other methods (Miles’ operation, *n *= 16; Hartmann’s operation, *n *= 8; total pelvic extirpation, *n* = 3; transanal operation, *n *= 17; and others, *n *= 6). A total of 136 patients with primary rectal cancer were finally included in this study (Fig. 1). Preoperative chemotherapy or chemoradiotherapy was administered for complex cases, such as bulky tumors or tumors with extramural invasion [18]. Ten patients underwent preoperative chemotherapy, and two patients underwent chemoradiotherapy (Table 1).

**Table 1:** Patient characteristics

Variables	All (*n *= 136)	All (*n *= 136)		*p* value
		AL: − (*n *= 115)	AL: + (*n *= 21)	
AL: −/+	115/21 (84.6/15.4)			
Sex: Female/Male	55/81 (40.4/59.6)	52/63 (45.2/54.8)	3/18 (14.3/85.7)	0.016
Age†	68.0 [60.8, 74.0]	68.0 [61.0, 74.0]	65.0 [59.0, 69.0]	0.134
BMI†	21.4 [19.5, 23.7]	21.5 [19.4, 23.9]	21.1 [20.7, 23.4]	0.964
Section: Ra/Rb	60/76 (44.1/55.9)	53/62 (46.1/53.9)	7/14 (33.3/66.7)	0.399
NLR†	2.4 [1.8, 3.4]	2.3 [1.8, 3.3]	2.9 [2.4, 3.4]	0.075
LMR†	5.1 [3.5, 6.3]	5.2 [3.6, 6.4]	4.9 [3.2, 5.7]	0.293
Alb†	4.2 [3.9, 4.5]	4.2 [3.9, 4.5]	4.1 [3.7, 4.4]	0.242
Stoma: −/+	84/52 (61.8/38.2)	68/47 (59.1/40.9)	16/5 (76.2/23.8)	0.217
Drain: −/+	72/64 (52.9/47.1)	60/55 (52.2/47.8)	12/9 (57.1/42.9)	0.856
Operation time (min)†	348.5 [298.8, 453.2]	339.0 [295.0, 436.0]	445.0 [369.0, 489.0]	0.037
Blood loss (ml)†	53.0 [20.0, 210.0]	50.0 [20.0, 175.0]	190.0 [50.0, 573.0]	0.014
Tumor size (mm)†	35.0 [22.0, 50.0]	33.0 [20.0, 50.0]	60.0 [40.0, 70.0]	< 0.001
Laparotomy: −/+	122/14 (89.7/10.3)	106/9 (92.2/7.8)	16/5 (76.2/23.8)	0.043
UICC-T factor				0.213
T0	1 (0.7)	1 (0.9)	0 (0.0)	
Tis	2 (1.5)	2 (1.7)	0 (0.0)	
T1	33 (24.3)	31 (27.0)	2 (9.5)	
T2	36 (26.5)	32 (27.8)	4 (19.0)	
T3	60 (44.1)	46 (40.0)	14 (66.7)	
T4	4 (2.9)	3 (2.6)	1 (4.8)	
UICC-stage				0.137
0	1 (0.7)	1 (0.9)	0 (0.0)	
I	61 (44.9)	56 (48.7)	5 (23.8)	
II	16 (11.8)	12 (10.4)	4 (19.0)	
III	40 (29.4)	33 (28.7)	7 (33.3)	
IV	18 (13.2)	13 (11.3)	5 (23.8)	
Neoadjuvant treatment*: −/+	124/12 (91.2/8.8)	104/11 (90.4/9.6)	20/1 (95.2/4.8)	0.691
Lateral pelvis (mm)†	141.0 [135.0, 146.0]	141.0 [136.0, 146.0]	142.0 [132.0, 144.0]	0.400
Sacrum (mm)†	123.0 [114.0, 133.2]	123.0 [114.0, 132.5]	130.0 [119.0, 142.0]	0.029
Pelvic inlet (mm)†	115.0 [105.0, 122.0]	114.0 [105.0, 121.5]	115.0 [109.0, 123.0]	0.545
Pelvic outlet (mm)†	89.0 [84.0, 96.2]	89.0 [84.0, 98.0]	91.0 [83.0, 94.0]	0.411

Values in parentheses are percentages; †, median [interquartile range]; *, Neoadjuvant treatment includes neoadjuvant chemotherapy/chemoradiotherapy

*AL* anastomotic leakage; *BMI* body mass index; *NLR* neutrophil–lymphocyte ratio; *LMR* lymphocyte-monocyte ratio; *Alb* serum albumin; *Tis* tumor in situ

We examined the following variables that represented protective and risk factors for AL: sex, age, body mass index (BMI), tumor location (upper rectum [Ra] or lower rectum [Rb]), neutrophil-to-lymphocyte ratio (NLR), lymphocyte-to-monocyte ratio (LMR), serum albumin (Alb), UICC-T factor, UICC-stage, diverting stoma construction, use of an intraluminal drain, operating time, intraoperative blood loss, tumor size, and laparotomy. The clinical stage was classified preoperatively according to the UICC-TNM classification (8^th^ edition) and confirmed by postoperative histopathological examination findings.

The study was conducted in accordance with the ethical standards laid down in the 1964 Declaration of Helsinki and its later amendments and the ethical guidelines for clinical studies. The study protocol was approved by the institutional review board of Yamaguchi University (H28-186).

### Surgical technique

The standard surgical technique for most patients was laparoscopic low anterior resection, except for a few patients who underwent open midline laparotomy. Ligation and resection of the inferior mesenteric artery and vein were performed, followed by TME. Intersphincteric resection was carried out for very low rectal tumors. Bilateral lymph node dissection was performed in cases, where the main tumor location was lower than Rb and the invasion depth was deeper than T3 [19]. The double stapling technique (DST) or handsewn anastomosis type of neorectal reconstruction was performed. A temporary stoma was created at the surgeons’ discretion. A transanal drain was inserted in all patients in the latter half of the study period.

### Measurements of the pelvis

**Fig. 2:**
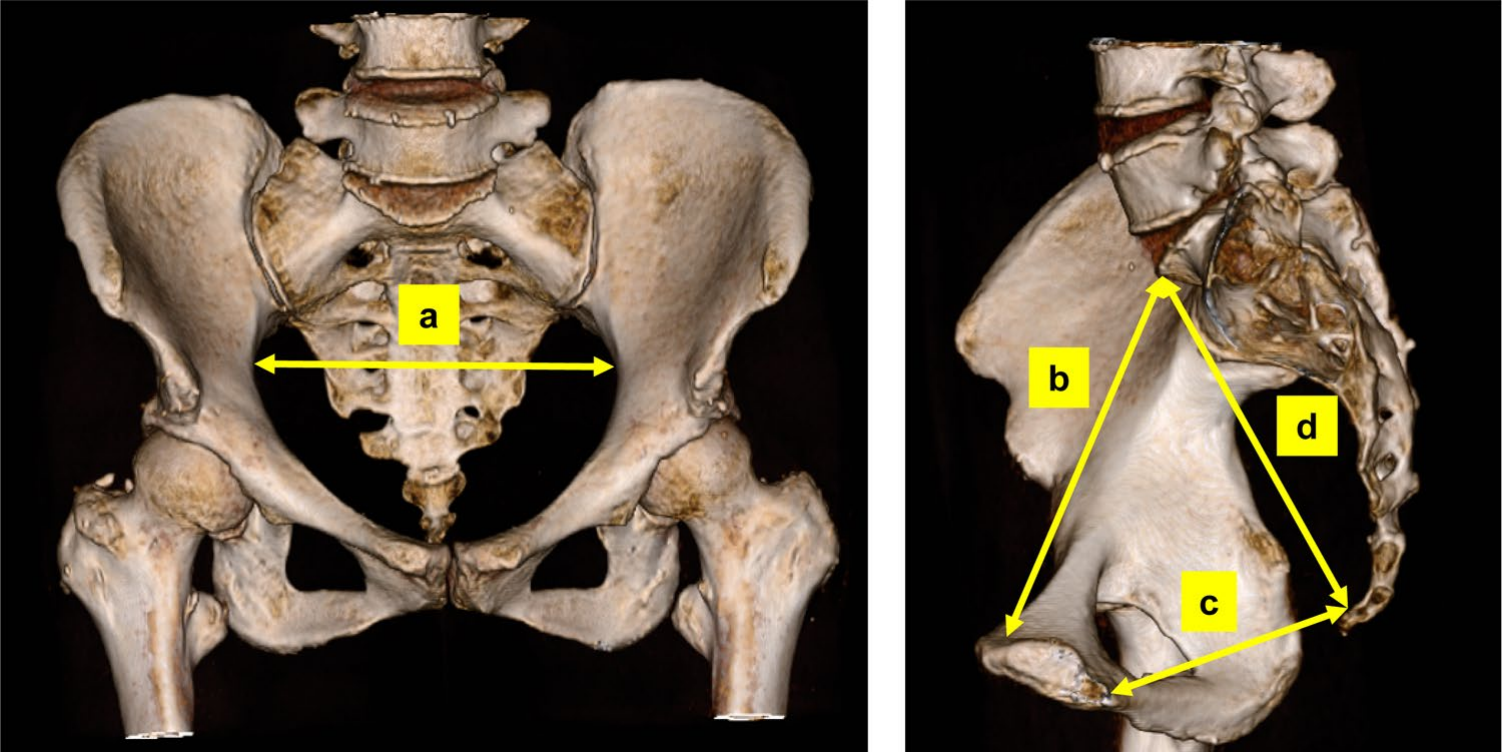
Measurements of pelvic dimensions. The four parameters were as follows: a = length of the lateral pelvis, b = length of the pelvic inlet, c = length of the pelvic outlet, and d = length of the sacrum

A pelvic CT examination was performed in all patients as a part of the routine preoperative work-up for rectal surgery. Using reconstructed coronal and sagittal CT images, a few examiners who were blinded to the patients’ information evaluated the following dimensions of the pelvis: a = length of the lateral pelvis, b = length of the pelvic inlet, c = length of the pelvic outlet, and d = length of the sacrum (Fig. 2). We used these parameters in further analyses.

#### Definition of anastomotic leakage

AL was detected based on the following clinical signs: discharge of pus or stool from the abdominal drain, presence of peritonitis with a high fever, tachycardia, abdominal pain, tenderness, or severe inflammation. The presence of an abscess, fluid collection, or free air surrounding the anastomotic site was checked for using CT to assess the presence of AL if any leak was suspected. However, asymptomatic AL was difficult to consider, as we did not perform contrast enemas routinely.

### Statistical analyses

The continuous and categorical variables were expressed as median values (with interquartile range [IQR]) and frequencies, respectively. For the comparisons of variables between the non-AL and AL groups, the Mann–Whitney *U* test was conducted for continuous variables, and the Chi squared test or Fisher’s exact test was used for categorical variables. A receiver operating characteristic (ROC) curve analysis using logistic regression was used to determine the cut-off values based on the point closest to (0, 1) criterion for each continuous variable. To identify the protective/risk factors for AL, logistic regression analyses were performed. Variables were included in Firth’s bias-reduced multiple logistic regression analysis with the stepwise Akaike’s Information Criterion (AIC) variable selection method when a *p* value < 0.20 was observed in the univariate analysis. The confidence intervals and *p* values for each factor were calculated by the Wald method.

All statistical analyses were performed using R language (R Core Team URL https://www.R-project.org/, Vienna, Austria). The logistic regression, ROC curve analysis, variable selection based on AIC, and Firth’s bias-reduced multiple logistic regression were conducted using stats::glm, pROC::cords [20], stats::glm, and logistf:logistf functions, respectively. A *p* value < 0.05 was considered statistically significant.

## Results

### Patient characteristics

Table 1 shows the characteristics of the patients in the study. In total, 136 patients underwent LAR with DST or handsewn anastomosis, including 81 (59.6%) males and 55 (40.4%) females. The median age was 68.0 years (IQR, 60.8–74.0 years) and their median. The median BMI was 21.4 kg/m^2^ (IQR, 19.5–23.7 kg/m^2^). Sixty patients (44.1%) and 76 patients (55.9%) had Ra and Rb cancer, respectively. Twenty-one patients (15.4%) developed AL, including 18 males and 3 females. The median BMI was 21.1 kg/m^2^ (IQR, 20.7–23.4 kg/m^2^); 7 of them (33.3%) had Ra cancer, and 14 (66.7%) had Rb cancer. We performed re-operation on 17 patients (81.0%). We constructed a covering stoma and performed intraabdominal lavage and drainage in 16 of those 17 patients. Four of the five patients with covering stomas were treated using drainage. However, we performed re-operation with intraabdominal lavage and drainage on the remaining patient. There were 4 patients (19.0%) with AL who did not need re-operation and were treated by drainage with an abdominal drain. The median post-surgery time until hospital discharge was 44 days (range 14–89 days). There were no deaths related to AL in this study. Among the 136 patients, 52 (38.2%) underwent construction of a covering stoma, and 14 (10.3%) underwent laparotomy. Five of those 14 patients developed AL.

### AL with or without a diverting stoma

We stratified patients based on the construction of a stoma, with the results, as shown in Table 2. Among the patients without stomas (*n *= 84), sex (*p *= 0.006), operation time (*p *< 0.001), blood loss (*p *= 0.024), and tumor size (*p *= 0.005) were the factors that showed significant differences between the AL (−) (*n *= 68) and AL (+) (*n *= 16) patients. Furthermore, Ra cancer was more frequent in AL (−) patients, while Rb cancer was more frequent in AL (+) patients (*p *= 0.051). Among patients who underwent construction of a stoma (*n *= 52), there were significant differences in intraoperative blood loss (*p *= 0.033), tumor size (*p *= 0.027), laparotomy (*p *= 0.004), UICC-stage (*p *= 0.015), and sacrum factors (*p *= 0.044) between AL (-) (*n *= 47) and AL (+) patients (*n *= 5).

**Table 2:** Patient characteristics stratified by stoma construction

Variables	Stoma: − (*n *= 84)		*p* value	Stoma: + (*n *= 52)		*p* value
	AL: − (*n *= 68)	AL: + (*n *= 16)		AL: − (*n *= 47)	AL: + (*n *= 5)	
Sex: female/male	37/31 (54.4/45.6)	2/14 (12.5/87.5)	0.006	15/32 (31.9/68.1)	1/4 (20.0/80.0)	1
Age†	68.0 [59.0, 75.2]	65.5 [59.5, 69.2]	0.198	68.0 [63.0, 71.9]	65.0 [59.0, 68.0]	0.446
BMI†	21.1 [19.2, 22.9]	21.4 [20.7, 23.4]	0.396	22.5 [20.2, 25.5]	20.7 [18.4, 21.1]	0.352
Section: Ra/Rb	46/22 (67.6/32.4)	6/10 (37.5/62.5)	0.051	7/40 (14.9/85.1)	1/4 (20.0/80.0)	1
NLR†	2.4 [1.8, 3.6]	2.9 [2.4, 3.2]	0.257	2.3 [1.7, 3.0]	3.4 [2.6, 3.9]	0.210
LMR†	5.3 [3.6, 6.6]	4.9 [3.2, 5.5]	0.415	4.8 [3.7, 6.2]	4.9 [3.2, 5.7]	0.555
Alb†	4.3 [4.0, 4.5]	4.2 [3.7, 4.4]	0.382	4.2 [3.8, 4.4]	3.8 [3.7, 3.9]	0.297
Drain: −/+	36/32 (52.9/47.1)	7/9 (43.8/56.2)	0.701	24/23 (51.1/48.9)	5/0 (100.0/0.0)	0.059
Operation time (min)†	304.5 [273.8, 339.0]	414.5 [320.8, 468.2]	< 0.001	466.0 [398.5, 619.0]	445.0 [419.0, 489.0]	0.733
Blood loss (ml)†	30.0 [15.0, 60.0]	112.5 [41.2, 540.0]	0.024	160.0 [38.5, 230.0]	1100.0 [190.0, 2365.0]	0.033
Tumor size (mm)†	30.0 [20.0, 50.0]	55.0 [38.8, 66.2]	0.005	35.0 [25.0, 50.0]	70.0 [55.0, 80.0]	0.027
Laparotomy: −/+	61/7 (89.7/10.3)	14/2 (87.5/12.5)	0.679	45/2 (95.7/4.3)	2/3 (40.0/60.0)	0.004
UICC-T factor			0.373			0.338
T0	0 (0.0)	0 (0.0)		1 (2.1)	0 (0.0)	
Tis	1 (1.5)	0 (0.0)		1 (2.1)	0 (0.0)	
T1	22 (32.4)	2 (12.5)		9 (19.1)	0 (0.0)	
T2	18 (26.5)	4 (25.0)		14 (29.8)	0 (0.0)	
T3	25 (36.8)	9 (56.2)		21 (44.7)	5 (100.0)	
T4	2 (2.9)	1 (6.2)		1 (2.1)	0 (0.0)	
UICC-stage			0.400			0.015
0	1 (1.5)	0 (0.0)		0 (0.0)	0 (0.0)	
I	35 (51.5)	5 (31.2)		21 (44.7)	0 (0.0)	
II	9 (13.2)	2 (12.5)		3 (6.4)	2 (40.0)	
III	18 (26.5)	6 (37.5)		15 (31.9)	1 (20.0)	
IV	5 (7.4)	3 (18.8)		8 (17.0)	2 (40.0)	
Neoadjuvant treatment*: −/+	68/0 (100.0/0.0)	16/0 (100.0/0.0)	1	36/11 (76.6/23.4)	4/1 (80.0/20.0)	1
Lateral pelvis (mm)†	142.0 [137.0, 148.0]	138.5 [131.8, 144.8]	0.168	140.0 [134.0, 144.0]	142.0 [132.0, 144.0]	0.804
Sacrum (mm)†	122.5 [114.0, 129.2]	128.0 [118.8, 140.0]	0.131	123.0 [113.0, 137.0]	145.0 [130.0, 151.0]	0.044
Pelvic inlet (mm)†	113.0 [105.0, 119.2]	115.5 [107.2, 123.2]	0.412	115.0 [105.5, 122.5]	115.0 [114.0, 115.0]	0.828
Pelvic outlet (mm)†	90.0 [84.0, 99.0]	89.0 [82.8, 94.5]	0.408	88.0 [84.0, 97.5]	91.0 [86.0, 92.0]	0.963

Values in parentheses are percentages; †, median [interquartile range]; *, Neoadjuvant treatment includes neoadjuvant chemotherapy/chemoradiotherapy

*AL* anastomotic leakage, *BMI* body mass index, *NLR* neutrophil–lymphocyte ratio, *LMR* lymphocyte-monocyte ratio, *Alb* serum albumin, *Tis* tumor in situ

### Univariate and multivariate analyses

**Fig. 3:**
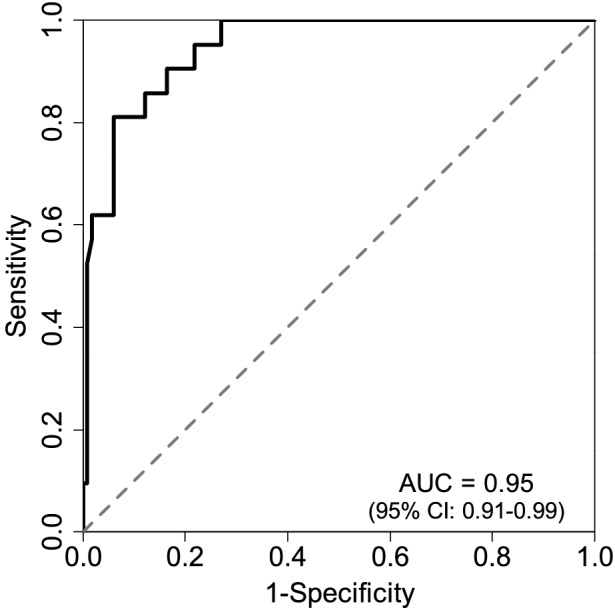
Model evaluation for AL. Firth’s bias-reduced multiple logistic regression analysis with the stepwise AIC variable selection method. The AUC was 0.95 (95% CI: 0.91–0.99), suggesting that the identified factors were strongly associated with AL. *AL* anastomotic leakage, *NLR* neutrophil–lymphocyte ratio, *LMR* lymphocyte–monocyte ratio, *Alb* serum albumin, *AIC* Akaike’s Information Criterion, *AUC* area under the curve, *CI* confidence interval

Finally, we checked for an association between the construction of a stoma and AL. Table 3 shows the results of univariate and multivariate logistic regression analyses. Firth’s bias-reduced multiple logistic regression analyses with the stepwise AIC variable selection method identified the variables of age (≥ 67 years), Alb (≥ 4 g/dl), and stoma (+) as protective factors and sex (male), NLR (≥ 2.4), LMR (≥ 5.3), operation time (≥ 373 min), blood loss (≥ 105 ml), tumor size (≥ 41 mm), and pelvic inlet size (≥ 113 mm) as risk factors for AL. The area under the curve (AUC) was 0.95 (95% confidence interval [CI]: 0.91–0.99), suggesting that these identified factors were strongly associated with AL (Fig. 3). Among the pre- and intraoperative factors, the construction of a covering stoma (stoma + , odds ratio [OR] 0.05, 95% CI [21] 0.01–0.26) was found to be a strong protective factor, and the operation time (≥ 373 min, OR 9.83, 95% CI 1.98–48.86), intraoperative blood loss (≥ 105 ml, OR 5.05, 95% CI 1.05–24.23), and pelvic inlet diameter (≥ 113 mm, OR 5.07, 95% CI 1.13–22.66) were identified as strong risk factors for AL from the multivariate analysis.

**Table 3:** Results of a logistic regression analysis

Variables	Univariate analysis				Multivariate analysis†			
	OR	95% CI		*p* value	OR	95% CI		*p* value
		Lower	Upper			Lower	Upper	
Sex: Male	4.95	1.38	17.74	0.014	3.85	0.92	16.09	0.065
Age (years) ≥ 67	0.43	0.17	1.11	0.081	0.39	0.11	1.44	0.157
BMI ≥ 21	1.03	0.40	2.62	0.958				
Section: Rb	1.71	0.64	4.55	0.283				
NLR ≥ 2.4	4.80	1.52	15.15	0.007	3.42	0.73	16.00	0.119
LMR ≥ 5.3	0.53	0.20	1.40	0.199	2.90	0.62	13.58	0.175
Alb ≥ 4	0.39	0.15	1.01	0.051	0.29	0.07	1.16	0.081
Stoma: +	0.45	0.16	1.32	0.146	0.05	0.01	0.26	< 0.001
Drain: +	0.82	0.32	2.09	0.675				
Operation time (min) ≥ 373	4.51	1.63	12.52	0.004	9.83	1.98	48.86	0.005
Blood loss (ml) ≥ 105	3.75	1.40	10.04	0.009	5.05	1.05	24.23	0.043
Tumor size (mm) ≥ 41	5.49	1.97	15.30	0.001	2.66	0.70	10.05	0.149
Laparotomy: +	3.68	1.09	12.38	0.035				
UICC-T: (T3, T4) vs. (T0, Tis, T1, T2)	3.37	1.22	9.30	0.019				
UICC-stage: (III, IV) vs. (0, I, II)	2.00	0.78	5.13	0.149				
Lateral pelvis (mm) ≥ 142	1.24	0.49	3.15	0.648				
Sacrum (mm) ≥ 129	2.81	1.09	7.26	0.033				
Pelvic inlet (mm) ≥ 113	2.06	0.75	5.70	0.162	5.07	1.13	22.66	0.034
Pelvic outlet (mm) ≥ 91	1.65	0.65	4.20	0.293				

†Variables were included in the Firth’s bias-reduced multiple logistic regression analysis with the stepwise AIC variable selection method when a *p*-value < 0.20 was observed in the univariate analysis

*AIC* Akaike’s Information Criterion, *BMI* body mass index, *NLR* neutrophil–lymphocyte ratio, *LMR* lymphocyte-monocyte ratio, *Alb* serum albumin, *Tis* tumor in situ, *OR* odds ratio, *CI* confidence interval

## Discussion

In our study, the overall rate of AL occurrence was 15.4% (21/136). In patients with stomal construction, the rate of AL occurrence was 9.6% (5/52), and in those without a stoma, the AL rate was 19.0% (16/84). In our institute, the rate of AL was nearly 10% in patients with covering stomas, which was markedly higher than that recorded in the Japanese database [22]. Regarding the consecutive patients that we evaluated in this study, there were 14 patients with laparotomy who developed AL significantly more frequently than laparoscopic procedure (*p *= 0.043), and among them, 3 of the 5 patients with covering stomas had AL. Regarding cases without laparotomy, there were 16 patients out of 122 (13.1%) and 2 out of 47 (4.3%) who had AL among all patients and those with stomas, respectively.

As we performed laparoscopic surgery in most cases, there were some cases with bulky tumors, and resection of these large lesions was difficult. There might have been some bias in the decision to construct a stoma, since it depended on the intraoperative findings. In other words, surgeons constructed a stoma in serious cases, so some reports found no relationship between AL and stoma construction [23, 24]. Our comparisons (Table 1) and a univariate analysis (Table 3) of the association between AL and stoma construction did not show any statistical significance. However, the multivariate logistic regression analysis showed that the construction of a covering stoma was a strong protective factor when other variables were held constant.

Figure 3 suggests that the AUC was 0.95 (95% CI 0.91–0.99), and the analyzed factors (i.e., sex, age, NLR, LMR, Alb, stoma, operation time, blood loss, tumor size, and pelvic inlet) were strongly associated with AL. A further analysis showed that the operation time (≥ 373 min), intraoperative blood loss (≥ 105 min), and pelvic inlet diameter (≥ 113 mm) were strong risk factors for AL (Table 3). Several risk factors were reported based on preoperative and intraoperative findings. A few studies identified risk factors for AL by measuring the patients’ pelvic dimensions using preoperative imaging examinations. According to those studies, the preoperative and intraoperative risk factors included the sex, operation time, and amount of intraoperative blood loss [13, 14].

Several studies have concluded that male sex is a risk factor for AL [24, 25]. Similarly, the findings of our analysis also indicated that males were at a significant risk of developing AL. Furthermore, the operation time and intraoperative blood loss were also reported as major risk factors for AL in many studies [13, 26]. Our analysis provided results that were comparable to these previous findings. The pelvic index was also reported as a risk factor in a previous study [15]. Tsuruta et al. reported that a smaller ratio of the mesorectum to the depth of the cavity of the lesser pelvis was associated with an increased risk of AL. We also observed that a larger pelvic inlet size (≥ 113 mm) was a risk factor when other variables were held constant. From our analysis considering other clinical confounding factors, we did not detect any confounders. The univariate analysis might have been affected by other confounding factors. However, the multivariate analysis reduced these possible effects by other confounding factors. Therefore, regarding the pelvic index, the significant risk factors differed between the univariate and multivariate analyses. A longer sacrum and pelvic inlet were significant risk factors for AL in univariate and multivariate analyses, respectively (Table 3).

Initially, we measured several pelvic dimensions (short lengths of the lateral pelvis, anteroposterior diameter, and sacrum) under the hypothesis that males, with their narrow pelvis, might have a higher risk of AL than females. However, our analysis showed that a longer pelvic inlet was a significant risk factor for AL. This result may be explained by the presence of a deeper pelvis making it difficult to perform the operation. In other words, surgeons considered that the narrow pelvis during the operation was due not only to the skeletal structure but also the soft tissue, such as the muscles, intestinal size, and amount of mesenteric adipose tissue.

This study is limited by its nature as a retrospective record review performed at a single institution. In the future, our findings should be confirmed in more cases.

## Conclusion

In conclusion, the results obtained after analyzing the data of 136 patients with rectal cancer suggested that the construction of a covering stoma was a possible protective factor. The results further indicated that a longer operation time, higher intraoperative blood loss, and larger pelvic inlet dimensions were possible risk factors for AL after low anterior resection in patients with rectal cancer. Further prospective studies may help clarify the pertinent factors predicting postoperative morbidity in patients with rectal cancer.

## Data Availability

The datasets generated during and/or analyzed during the current study are available from the corresponding author on reasonable request
